# The immune subtypes and landscape of sarcomas

**DOI:** 10.1186/s12865-022-00522-3

**Published:** 2022-09-24

**Authors:** Weiwei Weng, Lin Yu, Zhang Li, Cong Tan, Jiaojie Lv, I. Weng Lao, Wenhuo Hu, Zhenzhong Deng, Zebing Liu, Jian Wang, Midie Xu

**Affiliations:** 1 Department of Pathology, Fudan University Shanghai Cancer Center, Shanghai, 200032 People’s Republic of China; 2 Department of Oncology, Shanghai Medical College, Fudan University, Shanghai, 200032 People’s Republic of China; 3 Institute of Pathology, Fudan University, Shanghai, 200032 People’s Republic of China; 4 Department of Oncology, Xinhua Hospital, School of Medicine, Shanghai Jiao Tong University, Shanghai, 200092 People’s Republic of China; 5 Human Oncology and Pathogenesis Program, Memorial Sloan Kettering Cancer Center, New York, NY USA; 6 Marie-Josée and Henry R. Kravis Center for Molecular Oncology, Memorial Sloan Kettering Cancer Center, New York, NY USA; 7 Department of Pathology, Renji Hospital, School of Medicine, Shanghai Jiao Tong University, Shanghai, 200127 People’s Republic of China

**Keywords:** Sarcoma, Immune subtype, Immune landscape, Molecular characteristics, Immunotherapeutic response

## Abstract

**Background:**

Considering the molecular heterogeneity of sarcomas and their immunologically quiet character, immunotherapy (e.g., immune checkpoint inhibitors) plays a viable role in only a subset of these tumors. This study aimed to determine the immune subtypes (IMSs) of sarcomas for selecting suitable patients from an extremely heterogeneous population.

**Results:**

By performing consensus clustering analysis of the gene expression profiles of 538 patients with sarcomas in online databases, we stratified sarcomas into three IMSs characterized by different immune cell features, tumor mutational burdens (TMBs), gene mutations, and clinical outcomes. IMS1 showed an immune “hot” and immunosuppressive phenotype, the highest frequencies of CSMD3 mutation but the lowest frequencies of HMCN1 and LAMA2 mutations; these patients had the worst progression-free survival (PFS). IMS2 was defined by a high TMB and more gene mutations, but had the lowest frequency of MND1 mutations. IMS3 displayed the highest MDN1 expression level and an immune “cold” phenotype, these patients had the worst PFS. Each subtype was associated with different expression levels of immunogenic cell death modulators and immune checkpoints. Moreover, we applied graph learning-based dimensionality reduction to the immune landscape and identified significant intra-cluster heterogeneity within each IMS. Finally, we developed and validated an immune gene signature with good prognostic performance.

**Conclusions:**

Our results provide a conceptual framework for understanding the immunological heterogeneity of sarcomas. The identification of immune-related subtypes may facilitate optimal selection of sarcoma patients who will respond to appropriate therapeutic strategies.

**Supplementary Information:**

The online version contains supplementary material available at 10.1186/s12865-022-00522-3.

## Background

Sarcomas, which account for less than 1% of primary malignancies, comprise a heterogeneous group of tumors derived from mesenchymal tissues. Sarcomas can be further divided into more than 100 subtypes, as defined by the World Health Organization based on distinct morphological and genetic changes [1]. Although most sarcomas are detected as local tumors and treated with surgery and radiation, the heterogeneity of their cellular composition results in a complicated prognosis. Indeed, different histologic types have different presentations, behaviors, and outcomes, with 50% being fully malignant and often metastatic and 50% being locally aggressive with some but limited metastatic potential [2]. According to European Society for Medical Oncology (ESMO) and National Comprehensive Cancer Network (NCCN) guidelines, chemotherapy is the cornerstone of traditional treatment for advanced and metastatic sarcoma [3–6]. However, even front-line chemotherapy is associated with limited objective response rates (ORRs, averaging ~ 18%) [7, 8]. Given that an increased cure rate due to specific systemic therapies have been achieved for only a few sarcoma subtypes [9, 10], effective systemic treatment for advanced sarcoma is still an unmet need.

Considering the rapid advancements and remarkable survival benefits in patients with various tumors, immunotherapy is now considered to be the fifth pillar of antitumor therapy, after surgery, chemotherapy, radiation and targeted therapy [11, 12]. Although sarcomas are generally considered immune “cold” tumors, with a low level of immune infiltration, numerous attempts have been made to use immunotherapy to treat sarcomas [13–15]. Nevertheless, immunotherapy is only a viable treatment approach for certain subsets of sarcomas. A recent systematic review and meta-analysis reported that treatment with immune checkpoint inhibitors (ICIs) led to significantly higher response rates in classic Kaposi sarcoma (CKS), alveolar soft part sarcoma (ASPS) and undifferentiated pleomorphic sarcoma (UPS). Conversely, low or no response has been found for gastrointestinal stromal tumors (GISTs), desmoplastic small round cell tumors (DSRCTs), endometrial stromal sarcomas, epithelioid hemangioendotheliomas, malignant peripheral nerve sheath tumors (MPNSTs), myxoid liposarcomas (MLPSs) and spindle cell sarcomas. Therefore, the clinical activity of ICIs in sarcomas is highly variable [16]. Overall, the positive response of a substantial proportion of sarcoma patients to immunotherapy suggests that more efforts should be made to determine which patients are most likely to respond. Manipulation of immune regulatory pathways has been proven to be effective in different subsets of sarcomas with paradigmatic immune-sensitive/“hot” tumors, which harbor high levels of tumor mutational burden (TMB), CD8^+^ lymphocytes and programmed death-ligand 1 (PD-L1) expression [17–19] and are thus sensitive to ICIs. Scientists have also applied various approaches, such as messenger RNA vaccines, to reprogram the tumor microenvironment to increase immune-mediated responses, aiming to switch “cold” tumors to “hot” tumors [20]. Attempts to manipulate the microenvironment of sarcomas are also underway. Sarcomas are traditionally considered immunologically quiet tumors because of the low immunosuppressive tumor environment (TME) and TMB (< 10 mutations/Mb) in the majority of cases [17], with dMMR/MSI-H only found in 0–4% of sarcomas [21]. Interestingly, there is evidence that antigen presentation in sarcomas can be altered. For example, destruction of tumor cells by local injection of oncolytic viruses promotes release of tumor-associated antigens (TAAs) that prime the immune system, thereby promoting a more effective systemic antitumor immune response in locally advanced or metastatic sarcoma [22]. Several recent clinical studies have provided solid evidence that the ORR can be improved by enhancing antitumor immunity with combination approaches such as ICIs together with radiotherapy or systemic therapy [23]. Given the TME of sarcoma, most histological subtypes may require a multipronged approach to manipulate the microenvironment and thus induce effective antitumor immunity. Regardless, which kind of TME in sarcoma accounts for the response rate to immunosuppressant’s remains largely unknown.

Genetically, human sarcomas are classified based on the abnormalities that drive their pathogenesis, and sarcomas can be categorized into two main groups: (1) sarcomas with simple and specific chromosomal changes (often translocations) and a low mutational burden and (2) karyotypically complex sarcomas with numerous copy number aberrations and a moderate mutational burden [24]. Among these copy number-driven tumors, UPS, dedifferentiated liposarcoma (DDLPS) and—to a lesser extent—leiomyosarcoma (LMS) can exhibit durable responses to immune checkpoint inhibitors, possibly due to heterogeneity within the tumors [25, 26]. However, translocation-associated subtypes, synovial sarcoma (SS), and MLPS tend to show mixed results [24]. The biological features of these two groups have suggested the possibility of distinct immune subtypes that cause differences in overall clinical outcomes. It should be noted that the category is broadly based on location and morphology and does not reflect the immune microenvironment of a particular sarcoma and therefore does not provide molecular evidence for which specific types of sarcomas may benefit from immunotherapy.

Here, we present a multicohort retrospective study and classify sarcomas into three distinct immune subtypes (IMSs) based on consensus clustering of immune-related gene expression profiles. We further demonstrate the stability and reproducibility of this classification in an independent cohort. Each of the three immune subtypes was found to be associated with distinct molecular and cellular features and clinical outcomes. Identification of immune-related subtypes may facilitate optimal selection of sarcoma patients responsive to immunotherapy. Finally, we identified six immune gene modules and selected five genes from the most prognosis-related module to develop and validate an individualized gene set-based prognostic signature for sarcoma.

## Results

### Immune subtypes in sarcoma

**Fig. 1:**
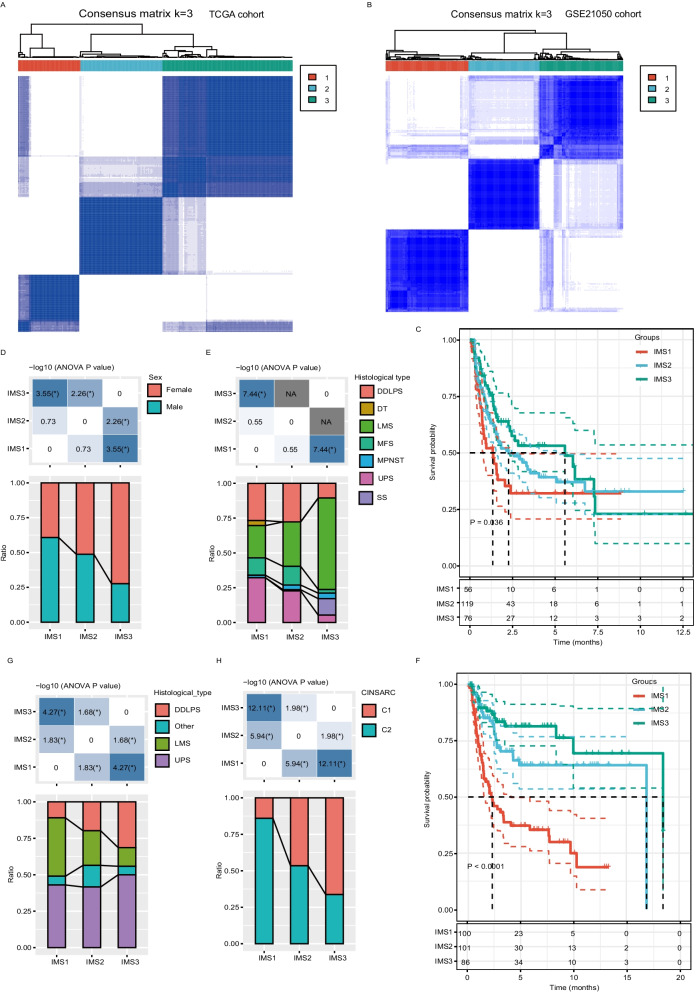
Identification of potential immune subtypes of sarcoma. **A** Sample clustering heatmap of the 251 samples in the cohort from *TCGA*. **B** Sample clustering heatmap of the 287 samples in the cohort from GEO. **C** Kaplan–Meier curves with log-rank test showing the PFS of the sarcoma IMS in *TCGA*. **D, E** Distribution of IMS1-IMS3 across sarcoma (**D**) sex and (**E**) histological subtypes in *TCGA*. *P < 0.05. **F** Kaplan–Meier curves with log-rank test showing the PFS of sarcoma IMS in GSE21050. **G, H** Distribution ratio of IMS1-IMS3 across sarcoma (**G**) histological subtypes and (**H**) CINSARC subtypes in GSE21050. *P < 0.05. NA corresponds to data for which the chi-square test cannot be applied, such as a group of samples is 0. DDLPS, dedifferentiated liposarcoma; DT, desmoid tumor; LMS, leiomyosarcoma; MFS, myxofibrosarcoma; MPNST, malignant peripheral nerve sheath tumor; SS, synovial sarcoma; UPS, undifferentiated pleomorphic sarcoma

By performing consensus clustering for 251 sarcoma samples using the gene expression profile of 1914 annotated immune-related genes (IRGs) extracted from *The Cancer Genome Atlas* (*TCGA*), we identified three robust IMSs in the SARC cohort from the database (Fig. 1A, Additional file 1: Fig. S1). Of these identified ISs, IMS3 was associated with the best progression-free survival (PFS), whereas IMS1 was associated with the worst (Fig. 1B). In addition, we observed significant differences in the distribution of sex and histological subtype among the three IMSs. For example, male patients were preferentially in IMS1 and female patients in IMS3, and most LMSs and SSs were in IMS3 (Fig. 1C, D). We then assessed the reproducibility of the immune subtypes in the GSE21050 cohort to validate our findings in the SARC cohort from *TCGA*. Samples in the GSE21050 cohort were also assigned to three IMSs according to the same method. The obtained IMSs for the GSE21050 cohort displayed similar survival patterns (Fig. 1E), with significant differences in the distribution of histology among them (Fig. 1F). Nevertheless, the same IMS displayed the opposite distribution between DDLPS and LMS in the cohorts *TCGA* and GSE21050, indicating significant heterogeneity in these tumor types (Fig. 1G). Furthermore, we analyzed the relationship between IMSs and Complexity Index in SARComa (CINSARC) subtypes proposed by Frédéric Chibon and colleagues, which stratifies sarcoma prognosis into two subtypes (“low-risk, C1” and “high-risk, C2”) by using a 67-gene expression signature [27]. Consistent with the prognostic difference among the IMSs, the GSE21050 cohort results showed that IMS1 was strongly related to the high-risk C2 subtype and that IMS3 was associated with the low-risk C1 subtype (Fig. 1H). These data suggest that sarcoma can be classified into three IMSs with distinct characteristics.

### The relationship between IMS and TMB and common gene mutations

**Fig. 2:**
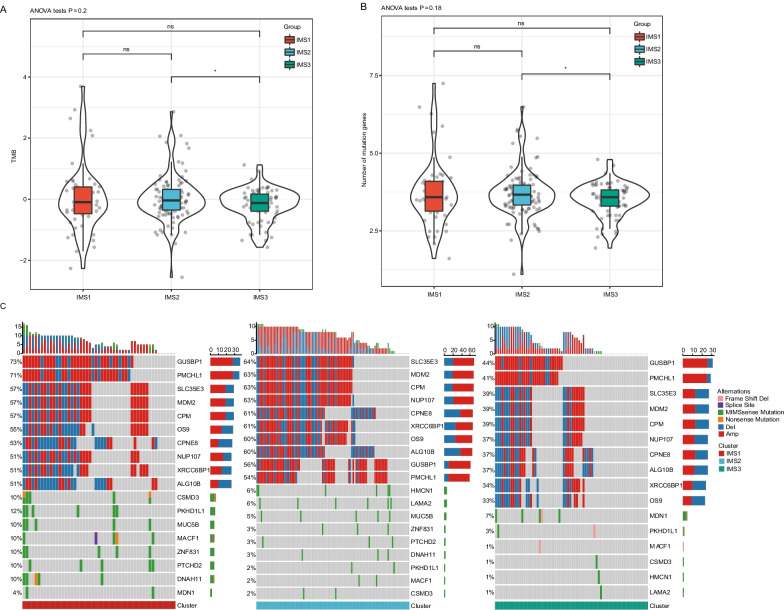
Association between immune subtype and TMB and gene mutation in the sarcoma dataset from *TCGA*. A-B TMB (**A**) and mutation (**B**) number in sarcoma IMS1-IMS3 in cohort from *TCGA*. Data are shown as the mean ± SD. *P < 0.05. **c** Top ten highly mutated gene copy number variation and genes in sarcoma immune subtypes

The TMB in the IMS2 group was significantly higher than that in the IMS3 group, whereas no significant difference was observed between IMS1 and IMS2 or between IMS1 and IMS3 (Fig. 2A). In addition, we counted the number of gene mutations in samples within each subtype and observed 549 genes with mutation frequency > 3 in all subtypes (Additional file 5: Table S2), and we identified 86 genes with significantly high mutation frequency in each subtype by using the chi-square test (P < 0.05, Additional file 6: Table S3). The number of gene mutations in the IMS2 group was significantly higher than that in the IMS3 group (Fig. 2B). The top 10 mutation characteristics with the highest mutation frequency in each subtype are shown in Fig. 2C. The proportion of CSMD3 mutations in the IMS1 group was significantly higher than that in the IMS2 and IMS3 groups, whereas the proportion of HMCN1 and LAMA2 mutations in the IMS1 group was significantly lower than that in the IMS2 and IMS3 groups; IMS2 had the highest proportion of HMCN1 mutation; and the proportion of MDN1 mutations in the IMS3 group was significantly higher than that in the IMS1 and IMS1 groups (Fig. 2C, Additional file 6: Table S3). The three IMSs showed different profile on gene amplification and deletion that GUSBP1 amplification was most common in IMS1 and IMS3, SLC35E3 amplification was most common in IMS2. Overall, IMS2 harbored the highest frequency of gene amplification and deletion (Fig. 2C). These data suggest that sarcoma samples in each IMS have distinct TMB and gene mutation characteristics.

### Expression of classic chemotherapy-induced immune response-related markers and immune checkpoint genes in each IMS

**Fig. 3:**
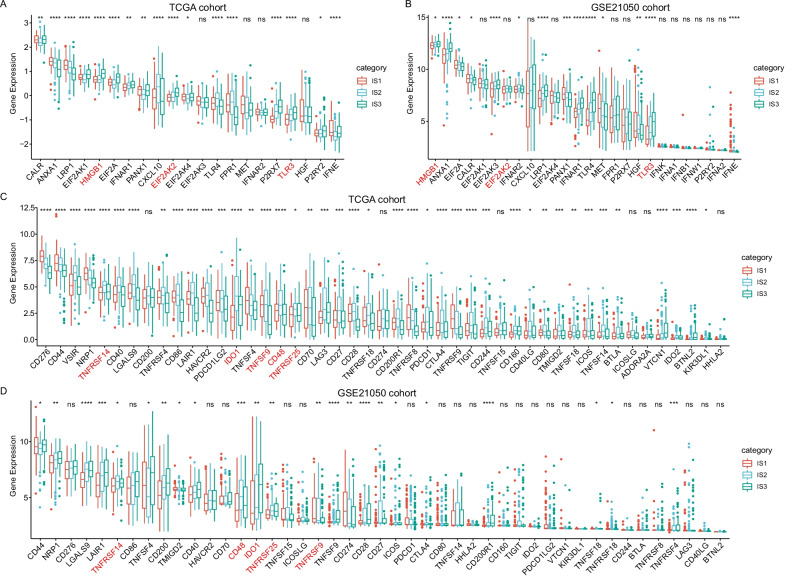
Association between immune subtypes and chemotherapy-induced immune response-related classic markers and immune checkpoint genes. **A**, **B** Differential expression of immune subtypes and classic chemotherapy-induced immune response-related markers among the sarcoma immune subtypes in the cohorts from *TCGA* (A) and GSE21050 (**B**). **C**, **D** Differential expression of immune checkpoint-related genes among the sarcoma immune subtypes in *TCGA* (**C**) and GSE21050 (**D**). The top and bottom of the box are the upper quartile (Q3) and the lower quartile (Q1) of the data, respectively. The solid line in the box represents the median. The whiskers represent the maximum and minimum values of this group of data. The Kruskal–Wallis test was used to assess significant differences. ns, not significant, *P < 0.01, **P < 0.001, ***P < 0.0001, and ****P < 0.00001

There were 21 chemotherapy-induced immune response-related classic genes expressed in the SARC cohort from *TCGA*. Among them, 17 (81%) genes showed significant differences between each subtype (Fig. 3A). In total, 26 genes were expressed in the GSE21050 cohort, with 14 showing significant differences between each subtype (Fig. 3B). The expression tendency of HMGB1, TLR3 and EIF2AK2 among the three IMSs was consistent between the cohorts *TCGA*-SARC and GSE21050, and all of them showed higher levels in IMS3 than in IMS1 and IMS2. Regarding the expression profile of 47 immune checkpoint-related genes, 41 (87.2%) genes showed significant differences among the three IMSs in the SARC cohort from *TCGA* (Fig. 3C); 23 of 45 immune checkpoint-related genes that could be detected in the GSE21050 cohort showed significant differences among the three IMSs (Fig. 3D). Of them, 21 genes displayed significant differences in both cohorts. Although their differences exhibited heterogeneity, IMS1 showed the lowest expression of IDO1, TNFRSF14 and TNFRSF25, IMS2 had the highest level of CD48, and IMS3 had the highest level of TNFSF9 in both cohorts (Fig. 3).

### Expression of tumor biomarkers in each IMS

**Fig. 4:**
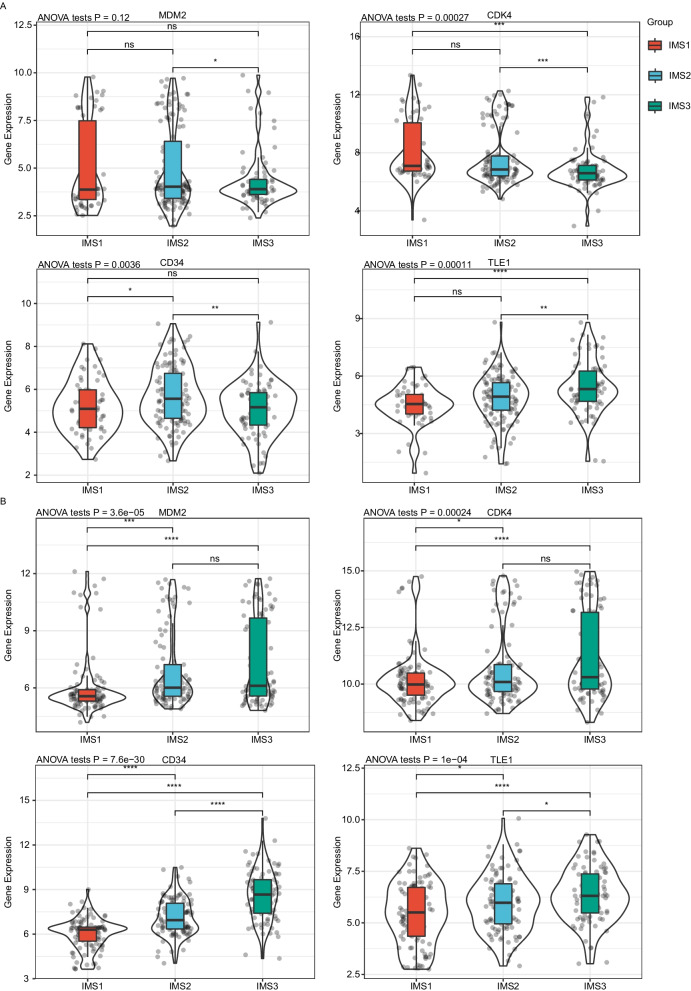
Association between immune subtypes and sarcoma-related tumor biomarkers. **A**, **B** MDM2, CDK4, CD34, and TLE1 expression in sarcoma immune subtypes in *TCGA* (**A**) and GSE21050 (**B**) cohorts. The solid black line in the box represents the median, and the black box in the violin plot represents the quartile range. The dots show the distribution of each sample, and the black vertical line running through the violin chart represents the interval from the minimum value to the maximum value. The Kruskal–Wallis test and Wilcox test were used to assess significant differences among the three groups and pairwise comparisons between groups, respectively. ns, not significant, *P < 0.01, **P < 0.001, ***P < 0.0001, and ****P < 0.00001

We extracted the expression profiles of four sarcoma-related tumor biomarkers, MDM2, CDK4, CD34, and TLE1, from the cohorts *TCGA*-SARC and GSE21050 and analyzed their expression profile in each IMS. CDK4, CD34, and TLE1 were significantly different among the three IMSs in the two cohorts, but only TLE1 showed consistent expression differences: its expression level in IMS3 was significantly higher than that in IMS1 and IMS2 (P < 0.0001, P < 0.05, respectively; Fig. 4). In addition, the CD34 expression level in IMS2 was significantly higher than that in IMS1 in both cohorts (both P < 0.05; Fig. 4). These results suggest a limited prediction accuracy of tumor biomarkers for sarcoma immunotype.

### Immune characteristics of IMSs

**Fig. 5:**
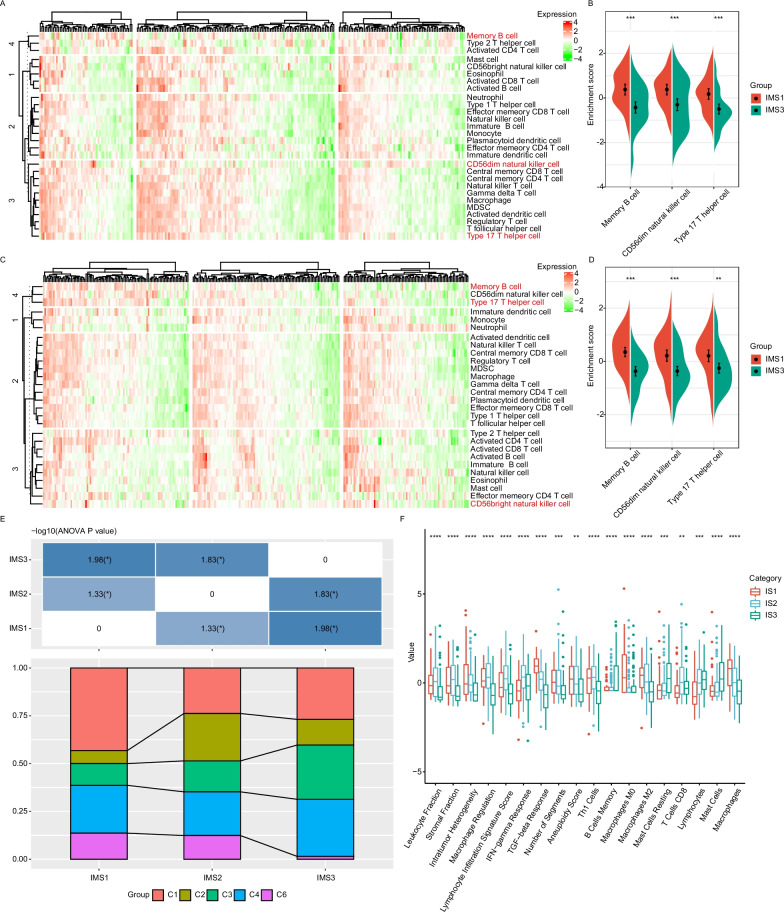
Cellular and molecular characteristics of immune subtypes. **A**, **C** Heatmap for the estimated enrichment scores of 28 immune cell signatures among sarcoma immune subtypes in *TCGA* (**A**) and GSE21050 (**C**) cohorts. Memory B cells, CD56dim natural killer cells and type 17T helper cells are highlighted in red. **B**, **D** Differential enrichment scores of memory B cells, CD56dim natural killer cells and type 17T helper cells in *TCGA* (**B**) and GSE21050 (**D**). **E** Distribution of sarcoma immune subtypes among 6 *TCGA* pancancer immune subtypes. IMS1 was mainly associated with to the C1 subtype, and the C2 subtype was mainly distributed within IMS2; the percentages of C3 and C4 subtypes in IMS3 were higher than those in IMS1 and IMS2. The percentage of the C6 subtype in IMS3 was lower than those in IMS1 and IMS2. **P < 0.01. **F** The estimated proportion of 18 significant immune-related features among immune subtypes with FDR < 0.01. The top and bottom of the box are the upper quartile (Q3) and the lower quartile (Q1) of the data, respectively. The solid black line in the box represents the median. The whiskers represent the maximum and minimum values of this group of data. The Kruskal–Wallis test was used to assess significant differences. **P < 0.001, *** P < 0.0001, and **** P < 0.00001

The immune characteristics of the IMSs in sarcoma could be represented by the distribution of 28 immune cell components (Fig. 5A–D). In the SARC cohort from *TCGA*, these immune cells were mainly divided into four immune categories (Fig. 5A). We also found the distribution of most of these immune cell components to differ among the three IMSs. For example, memory B cells, CD56dim natural killer cells and type 17T helper cells were significantly higher in IMS1 than in IMS3 (Fig. 5B), and this tendency also existed in the GSE21050 cohort (Fig. 5C, D), which suggests that the poor prognosis of IMS1 sarcoma may be related to activation of these cell types. We further compared the present results of IMSs with the previous six immune subtypes defined by *TCGA* pancancer study [28] and discovered that the identified IMS1 subtype mainly exhibited tendencies toward the C1, C4, and C6 subtypes; whereas the percentage of C6 subtypes in IMS3 was lowest among all six clusters (Fig. 5E). When assessing the correlation between IMS and 56 previously defined immune molecular characteristics [28], we identified the 18 most significant immune-related features using a false discovery rate (FDR) < 0.01 (Fig. 5F). Notably, IMS1 showed the highest macrophage infiltration among the three IMSs. However, the percentage of protumor M2 macrophages tended to be highest in IMS1 compared with the other two IMSs. Additionally, IMS1 showed the highest transforming growth factor beta (TGF-β) response score (Fig. 5F). IMS3 tumors tended to have higher levels of resting mast cells and mast cells than IMS1 and IMS2 tumors, yet IMS2 had the highest interferon-gamma (IFN-γ) response among the three IMSs. These results suggest that immune subtypes reflect the immune status of sarcoma.

### Immune landscape of sarcoma

**Fig. 6:**
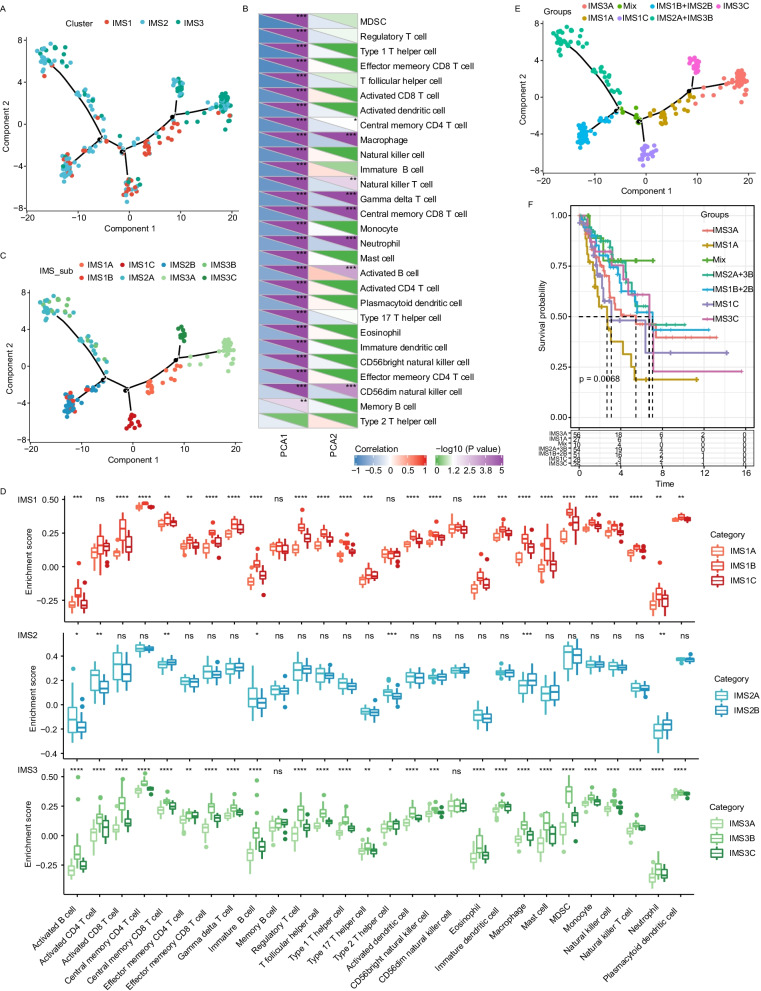
Immune landscape of sarcoma. **A** Immune landscape of sarcoma. Each point represents a patient, and the immune subtypes are color-coded. The horizontal axis represents the first principal component (PC1), and the vertical axis represents the second principal component (PC2). **B** Heatmap for the estimated enrichment scores of 28 immune cell signatures in two principal components. The lower part of each cell represents the R value of the correlation, the upper part represents the P value of the correlation, and significant cells have a significant mark (asterisk). Positive and negative correlations are located to the left and right of the 0 axis, respectively. For the P value, the median value of the division is 1.3 (i.e., − log10(0.05)). If the P value < 0.05, the corresponding color gradient is purple, and the green gradient represents a P value > 0.05. **C** Immune landscape of the subsets of each sarcoma immune subtype. **D** The estimated enrichment scores of 28 immune cell signatures in the above subsets. **E** Immune landscape of samples from three extreme locations and their prognostic status. **F** Kaplan–Meier curves showing the PFS of sarcoma immune subtypes in the SARC cohort from *TCGA*

Next, we gained insight into immune features in sarcomas by performing dimension reduction based on a graph-based learning approach. According to the results, all individual patients in the SARC cohort from *TCGA* were grouped into a manifold with sparse tree structures that defined the immune landscape of sarcoma (Fig. 6A). The location of individual patients in the five tree structures signified the comprehensive characterization of the tumor immune microenvironment in each distinct immune subtype. In fact, the horizontal coordinate (defined as principal component 1, PCA1) correlated highly with multiple immune cell modules, including MDSCs, regulatory T cells, type 1T helper cells, effector CD8^+^ T cells, T follicular helper cells, activated CD8^+^ T cells, activated dendritic cells, central memory CD4^+^ T cells, macrophages, and natural killer cells (r > 0.80), though the ordinate (defined as PCA2) was most relevant to macrophages and central memory CD8^+^ T cells (Fig. 6B). The integral distribution of each IMS was opposite to each other (Fig. 6A). Interestingly, we found that samples in IMS1 were distributed at the two horizontal opposite ends of the immune landscape; the same subtype also displayed an opposite distribution in IMS2 and IMS3, which suggests significant intracluster heterogeneity in IMS1. Consistently, we further divided IMS1 and IMS3 into three subtypes and IMS2 into two subtypes based on the location of immune cell populations (Fig. 6C); these subtypes showed different enrichment scores of immune cell characteristics (Fig. 6D). For example, IMS1A showed the lowest counts of B-cell and CD8^+^ T-cell proportions, regulatory T cells and MDSCs; IMS2B scored lower in terms of activated and immature B cells and activated CD4^+^ T cells, and IMS3B showed the highest counts of B-cell, CD4^+^ and CD8^+^ T-cell proportions (Fig. 6D).

Notably, IMS1A showed the lowest leukocyte and stromal fraction, macrophage regulation and lymphocyte infiltration signature score, and Th1 cell percentage among the three IMS1 subgroups. Although IMS1B had the highest macrophages regulation score, the percentage of protumor M2 macrophages tended to be highest in IMS1C compared with the other two IMS1 subgroups; and IMS1C had the highest wound healing score among the three subcategories of IMS1 (Additional file 2: Fig. S2A). Additionally, IMS2A showed higher CD8^+^ T cell percentage, while IMS2B showed the higher macrophages and macrophages M2 score (Additional file 2: Fig. S2B). Similar to IMS1A, IMS3A also showed the lowest leukocyte and stromal fraction, macrophage regulation and lymphocyte infiltration signature score, and Th1 cell percentage among the three IMS3 subgroups; IMS3B had the highest macrophages regulation score and tumor-suppressive M1 macrophages (Additional file 2: Fig. S2C).

Moreover, given that tumors under specific spatiotemporal conditions may have different prognostic characteristics, we performed prognostic comparison on samples in different distributional positions of the landscape and obtained 7 subgroups with different survival probabilities. Patients in IMS1A had the worst survival probability (Fig. 6E, F). Taken together, the immune landscape can be used to define the immune components of each sarcoma patient and predict their prognosis. Moreover, the immune landscape may help in selecting targeted regimens for different subtypes of patients and even facilitating selection of individualized therapeutic regimens for mRNA vaccines.

### Identification of immune gene coexpression modules and prognostic immune hub genes of sarcoma

**Fig. 7:**
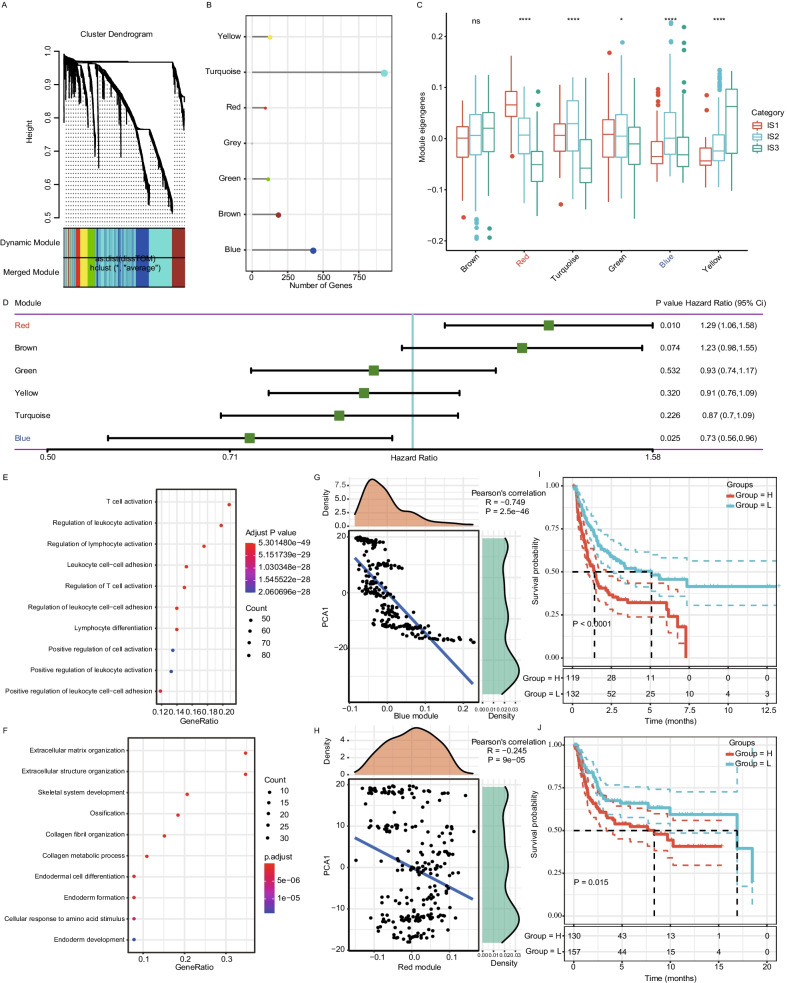
Identification of immune gene coexpression modules and prognostic immune hub genes of sarcoma. **A** Dendrogram of all differentially expressed genes clustered based on a dissimilarity measure (1-TOM). **B** Gene numbers in each module. **C** Differential distribution of feature vectors of each module in sarcoma subtypes. ns, not significant, *P < 0.01 and ****P < 0.00001. **D** Forest maps of univariate Cox regression survival analysis on the PFS of 6 modules in sarcoma. **E**, **F** Dot plot showing the top 10 GO terms in the blue (**E**) and red (**F**) modules. The dot size and color intensity represent the gene count and enrichment level, respectively. **G**, **H** Correlation between the blue (**G**) and red (**H**) module feature vectors and PC1 in the immune landscape. **I**, **J** Kaplan–Meier curves with log-rank test showing the differential PFS in the 5-gene signature with high and low risk in the datasets *TCGA* (**I**) and GSE21050 (**J**)

We clustered the 251 samples in the dataset from *TCGA* based on expression of 1914 immune-related genes (Additional file 3: Fig. S3A) and screened the coexpression gene module by setting the soft thresholding power to 3 (Additional file 3: Fig. S3B). To ensure that the coexpression network is a scale-free network, coexpression modules were screened by setting soft threshold power *β* to 10 (Additional file 3: Fig. S3C). A total of 7 coexpression modules (gray modules represent gene sets that could not be merged) were obtained after module fusion (Fig. 7A, B, Additional file 7: Table S4); among them, the turquoise modules had the highest number of genes (Fig. 7B). We further evaluated the distribution of the feature vectors of these 6 modules in each IMS and detected significantly different distributions in the 6 modules. The eigengenes of IMS1 in the yellow and blue modules were significantly lower than those of IMS3, whereas the eigengenes of IMS1 in the turquoise, green and red modules were significantly higher than those of IMS3 (Fig. 7C).

In survival analysis, we found that the blue and red gene modules correlated significantly with the prognosis of sarcoma in the dataset from *TCGA* (Fig. 7D). A low score in the blue module indicated a good prognosis, whereas a high score in the red module indicated a poor prognosis. Functional enrichment analysis of the blue and red modules showed that the blue module correlated with immune processes such as T-cell activation, regulation of leukocyte activation, and regulation of lymphocyte activation. (Fig. 7E) and that the red module correlated with the function of nonimmune cells, such as extracellular matrix organization and structure organization (Fig. 7F). The blue module correlated highly negatively with PC1 in the immune landscape (Fig. 7G); the red module correlated slightly negatively with PC1 in the immune landscape (Fig. 7H). Then, we extracted genes correlating with the module feature vector (coefficient greater than 0.75) in the blue module from the SARC dataset from *TCGA*. By univariate Cox proportional hazard regression analysis, six genes (C16orf54, CCR8, CXCR3, CYTIP, SAMD3 and TBX21) were found to be significantly associated with patient PFS (P < 0.05) (Additional file 8: Table S5). Furthermore, we performed stepwise regression analysis by using the stepAIC method in the MASS package based on the AIC Akaike information criterion and reduced the six genes to five (C16orf54, CCR8, CYTIP, SAMD3 and TBX21). Thus, we obtained a five-gene prognostic signature by multivariate survival analysis, and the formula was as follows: RiskScore = − 0.4270407 × expression ^C16orf54^ + 0.4710490 × expression ^CCR8^ + 0.3464125 × expression ^CYTIP^ + 0.4734728 × expression ^SAMD3^ − 0.5144081 × expression ^TBX21.^

We calculated the risk score of each sample and calculated the Z score for the risk score. After Z score transformation, samples with risk scores greater than zero were classified into a high-risk group (n = 119) and, samples less than zero into a low-risk group (n = 130). Kaplan–Meier curves showed an extremely significant difference between the two groups in *TCGA* dataset (P < 0.0001, Fig. 7I), and a consistent tendency was found in the GSE21050 dataset (P = 0.015, Fig. 7J). We selected these five genes as the final module feature genes; these hub genes can act as biomarkers for screening high-risk SARC populations and for identifying suitable populations for immunotherapy.

## Discussion

Sarcomas are very heterogeneous tumors, with more than 100 histologic subtypes characterized by the evolving recognition of distinct morphological and genetic features [1]. It is reported that the incidence of sarcoma has been increasing over time, but the efficacy of current systemic treatment options is limited [29, 30]. Nearly 40–50% of patients develop locally recurrent or metastatic disease within 5 years, and the median 5-years survival rate of patients with metastatic disease has remained low [31, 32]. It is thus critical to investigate novel therapeutic targets to apply new treatments with improved clinical efficacy. Immunomodulation in different forms, such as checkpoint blockers, tumor vaccines, and CAR-T cells, has become an area of interest for many tumors, and sarcoma is no exception [23]. However, sarcomas are thought to be “cold” tumors that show initial resistance to ICIs due to the lack or paucity of tumor T-cell infiltration [23]. Furthermore, sarcomas tend to show frequent overexpression of major suppressive cytokines such as vascular endothelial growth factor (VEGF) [33] or TGF-β1 [34], constituting hostile TME resistance to immune therapies. Due to the vast molecular heterogeneity of sarcomas and their immunologically quiet character, early studies based on clinical practice or ongoing trials with immunotherapies have shown limited effectiveness in of sarcoma treatment [13, 14, 25]. In general, sarcomas are not sufficiently immunogenic to trigger or sustain an immune response to generate tumor-specific immune effector cells. However, recently emerging evidence of ICI-based clinical trials is shedding new light on immunotherapies for sarcoma. For example, the SARC 028 trial in advanced sarcoma (NCT02301039) suggests that a B-cell rich feature and the presence of tertiary lymphoid structures are the TME basis for response to pembrolizumab in patients with UPS [35]. Patients who respond to ICIs had significantly higher infiltration of T-cells (CD8+, CD3+, PD-1+) and an increased percentage of tumor-associated macrophages expressing PD-L1 at baseline compared to nonresponders [19]. We are beginning to realize that the response of sarcoma to ICIs is more dependent on complex tumor-immune interactions than on tumor histology. For example, CKS lesions are characterized by variable proportions of spindle-shaped tumor cells, vessels, chronic inflammatory cells, and extravasated red blood cells. In general, they lack significant mitotic activity, cytologic pleomorphism, and necrosis, while the spindle cells often stain positively with vascular marker, CD31, CD34, and FLI-1, as well as lymphatic endothelial markers, such as D2-40, by immunohistochemical staining [36]. Early lesions (plaque or patch) often appear as a granulation type reaction with immune cell infiltration, intense angiogenesis, and proliferating “spindle”-shaped cells of endothelial and macrophagic cell origin, which are the tumor cells of Kaposi sarcoma [37]. UPS, used as a synonym for malignant fibrous histiocytoma (MFH), may still represent a heterogeneous group of lesions with variable outcomes, but differing progenitor cells and complex karyotypes, modern genomic analysis and immunohistochemical staining techniques have allowed for the term to be more narrowly defined [38]. Of note, T-cell infiltration and PD-L1 expression were found to be higher in sarcomas with complex genomics and particularly in UPS than in other soft tissue sarcomas [39]. DSRCT shows a poorly differentiated histologic appearance. On the basis of the poorly differentiated morphology, desmoplastic stroma, and desmin and cytokeratin immunoreactivity, as well as a positive *EWSR1-WT1* rearrangement study, DSRCT is diagnosed. Interestingly, DSRCT showed PD-1 on tumor cells instead of on tumor infiltrating lymphocytes [40, 41]. Hence, a better understanding of the sarcoma-specific immune microenvironment is essential for developing effective immunotherapies to improve the response and prognosis of patients.

In the current study, we performed comprehensive characterization of the immunological profile of sarcomas. We found that sarcomas could be stratified into three IMSs based on consensus clustering of immune-related gene expression profiles in *TCGA*-SARC data, and the reproducibility of this classification was demonstrated in the GSE21050 cohort as the validation cohort. Each of the three IMSs was associated with distinct genetic aberrations, tumor-infiltrating immune cell composition and functional orientation (immune “hot” and immune “cold”), and cytokine profiles, as well as different clinical outcomes. Among these four genes, CSMD3 mutation has been reported in DDLPS [42] and SS [43], and its mutation was identified as deleterious in DDLPS [42]. Although MDN1, HMCN1 and LAMA2 mutation were universally present and significantly different among the three groups, up until now there was no study had reported the mutation of these three genes in any kind of sarcoma. Our results suggest a need for individualized exploration on the clinical correlation of these mutations with each kind of sarcoma. Furthermore, given that the four biomarkers, MDM2, CDK4, CD34, and TLE1, are very helpful in the differential diagnosis of the histological types of sarcomas in clinical work, we analyzed the expression of the four molecules in the three IMSs to identify whether the four molecules also have differential diagnosis value for the immune subtypes of sarcoma. Our results show that from the perspective of immunophenotyping, these four commonly used biomarkers showed a limited prediction accuracy for sarcoma immunotype.

The IMS1 subtype was associated with the worst outcome while showing composite signatures reflecting a high immune cell component, such as memory B cells, CD56dim natural killer cells and type 17T helper cells, which suggests that the poor prognosis of sarcoma may be related to activation of these cell types. In addition, IMS1 showed the lowest IFN-γ response and CD8^+^ T cell and lymphocyte components among the three IMSs. These data suggest that the TME of IMS1 is immune “hot” but inflammation suppressive. Compared with the other two IMSs, IMS3 had the lowest TMB load and lowest immune cell component, two characteristics of immunologically quiet tumors. Moreover, IMS3 had the lowest lymphocyte infiltration and the lowest proportions of leukocytes, stromal cells, and macrophages. Thus, IMS3 corresponds to an immunologically “cold” tumor. Nevertheless, IMS3 has the lowest macrophage M2 component compared with the other IMSs, which may be attributed to the prolonged survival of patients in the IMS3 group [44–46]. The IMS2 group showed a relatively moderate immune microenvironment and clinical outcome. In addition, these immune subtypes may have intraclass heterogeneity. Further graph learning-based dimensionality reduction revealed intracluster heterogeneity in IMS1. A fraction of patients in IMS1 (IMS1A) showed significantly worse survival than others, which may mainly be attributed to the relative immune-suppressive TME with the least immune cell infiltration among all subclusters. Thus, mRNA vaccines may be relatively viable and more effective in IMS1A. In these IMS1A patients, combining immunotherapy with other local or systemic chemotherapy regimens to convert “cold” tumors to “hot” tumors might modulate both the host immune response and tumor microenvironment toward a state that is more conducive to therapy. Indeed, patients with the IMS2 and IMS3 subtypes had very similar prognoses. Overall, patients in IMS3B had the highest number of B cells, which may be the main contributor to their significantly better survival [47]. Notably, integrating the results of both immune subtypes and the immune landscape of sarcoma is important. From the perspective of mRNA vaccine application, patients with IMS1 tumors with higher TMB and somatic mutation rates may be more responsive to mRNA vaccines. In addition, the intracluster heterogeneity of each IMS and individual patients is informative for narrowing down the appropriate population for mRNA vaccines.

Compared with the previously defined pancancer immune subtypes based on data compiled by *TCGA* [28], IMS3 demonstrated the highest percentage of the C3 (inflammatory) subtype, which also had the best survival in the cohort from *TCGA*. Overall, the IMS1 subtype was mainly associated with the C1 (wound healing), C4 (lymphocyte depleted), and C6 (TGF-β dominant) subtypes, associated with a controversial phenotype with increased immune cell infiltration and a relatively better prognosis (C1), as well as an immunologically suppressed feature with a poorer prognosis (C4 and C6). Nevertheless, IMS1 can be further divided into three subcategories, in which immune cell infiltration in IMS1A tumors, which have the worst prognosis, is significantly lower than that in IMS1B and IMS1C tumors. In addition, IMS1A has the lowest lymphocyte infiltration signature score among the three subcategories of IMS1. Therefore, the C4 subtype of *TCGA* appear to mainly correspond to IMS1A. Moreover, IMS1C has the highest wound healing score among the three subcategories of IMS1, indicated that IMS1C was mainly composed of *TCGA* C1-like samples. To some extent, our immune landscape analysis results explain the contradiction between the IMS and *TCGA* pancancer immune subtypes and provide a more accurate subtyping method from the perspective of the immune TME. C2 is a subtype showing enrichment in many immune-evasion-related genes and high CD8^+^ T-cell infiltration, and our data demonstrate the C2 subtype was one of the mainly clusters distributed in IMS2. Consistently, both C2 and IMS2 exhibited intermediate survival. The C6 subtype, with an immunologically suppressed feature and the poorest prognosis of all six subtypes, displays the highest TGF-β signature and a high CD4 + T-cell infiltrate and is minimally distributed in IMS3. These results indicate that the three COAD IMSs map to different *TCGA* pancancer categories with similar immune microenvironments and that sarcoma is associated with immune subtypes different from the previously identified pancancer categories. Overall, our results may provide a useful and additional complement for the classification of the tumor immune microenvironment. Moreover, we observed that IMS3 is highly enriched in the low-risk C1 subtype of the CINSARC classification [27], suggesting that IMS3 is a highly conserved molecular subgroup in CINSARC C1. In addition, we observed a high proportion of CINSARC C2 samples in IMS1. This suggests the IMS1 samples to be mainly composed of conserved samples from CINSARC C2 and C2-like samples from CINSARC C1. The CINSARC C1 and C2 ratios were also similar in IMS2, suggesting an intermediate morphological molecular characteristic in addition to the two CINSARC subtypes. These results suggest that the IMS signature can be used as a complement to CINSARC classification.

In addition to prognostic prediction, immunotyping is indicative of therapeutic response to immunotherapies, and patients with different IMS phenotypes should respond differently to different treatment strategies. Theoretically, the immune “cold” phenotype (IMS3) may be related to many possible issues at different steps of the antitumor immune cycle, such as lack of tumor antigens, defect in antigen-presenting cells (APCs), absence of T-cell activation and deficit of homing into the tumor bed, leading to the absence of T-cell infiltration [48]. Therapeutic strategies such as demethylating agents [49], chemo-/radiotherapy inducing immunogenic cell death (ICD) [50], and tumor vaccines [51] might induce immune infiltration to reinvigorate the immune system in these patients. Newman and colleagues [20] recently demonstrated that application of the seasonal influenza vaccine to a cold tumor facilitates a shift toward a warm tumor. These findings are highly instructive for algorithms for the treatment of patients with IMS3 tumors. Inflamed phenotypes (IMS1 and IMS2) with higher TMB and somatic mutation rates are supposed to have greater responsiveness to immunotherapy and better prognosis. Nevertheless, given that the datasets we applied in the present study were not stratified by prior chemotherapy exposure and that there are no data suggesting that these signatures ultimately correlate with the response to immune therapy, there is some bias in our results, and the specific immunotherapy response of each IMS has yet to be confirmed in real-world clinical trials. However, the prognosis of patients with IMS1 was significantly poorer than that of patients with the other subtypes, suggesting that patients with this subtype are at high risk and require early intervention in postoperative immunotherapy. The critical factor determining prognosis might be the dominance of the immune-suppressive environment or the stimulatory environment. Smolle et al. [26] investigated relationships between tumor-infiltrating immune cells and patient/tumor-related factors in soft tissue sarcomas and assessed their prognostic value. They found that high macrophage levels were associated with increased local recurrence risk, irrespective of margins, age, sex or B-cell level.

We further performed weighted gene correlation network analysis (WGCNA), which stratified immune‐related genes into 7 modules. The distribution of the feature vectors of these modules was distinct among the IMSs. The feature vectors of IMS1 were found to be significantly lower in the blue module but significantly higher in the red module. Further analysis revealed that the immune gene coexpression modules were closely with the prognosis and cellular function of sarcoma. Furthermore, we reveal five targetable antigens (C16orf54, CCR8, CYTIP, SAMD3 and TBX21) by identifying modules associated with the sarcoma immune landscape. These five genes are promising mRNA vaccine candidates. Their dysregulation is related not only to survival but also to activation of T cells and regulation of leukocyte and lymphocyte activation. Hence, these antigens play a critical role in the occurrence and development of sarcoma and can be directly processed to the T-cell activation pathway to induce immune attack. Although further clinical evaluation is needed, the potential of these tumor antigens to serve as targets for sarcomas has been consolidated in previous reports. For instance, expression of CCR8 and TBX21 is proven to serve as immunotherapeutic targets and prognostic biomarkers in various solid tumors, respectively [52–54].

In general, infiltration and activation of T cells and other immune cells in tumor tissues, as well as inhibition of immune-suppressive cells, largely determine the therapeutic potential of mRNA vaccines in cancer patients with specific immune subtypes. Accordingly, mRNA vaccines might not be suitable for patients with high expression of genes clustered into blue and green modules. Finally, three hub genes with > 90% relevance in the blue module were identified, including MAP4 K1, TBC1D10C and TRAF3IP3, which are potential targets for mRNA vaccines.

## Conclusion

In conclusion, this study provides a conceptual framework for a better understanding of the tumor-specific immune microenvironment of sarcoma. Our findings demonstrate the immunological heterogeneity within sarcomas and provide a theoretical basis for predicting patient prognosis. In addition, stratification of patients according to the IMS system is needed to identify suitable patients and design adequate therapeutic strategies to improve the efficacy of immunotherapy. Furthermore, this study identified specific molecules (C16orf54, CCR8, CYTIP, SAMD3 and TBX21) that can be used as predictors of the risk of progression and therapeutic targets for patients with sarcomas.

## Materials and methods

### Patients and datasets

We collected 538 primary sarcoma samples from two databases: *The Cancer Genome Atlas* (*TCGA*) database and the Gene Expression Omnibus (GEO) database (dataset GSE21050). The RNA‐seq data, somatic mutation data, and corresponding clinical information of patients with follow-up information were obtained from the SARC dataset of *TCGA* (n = 251) using the GDC-client tool (https://portal.gdc.cancer.gov/). Gene IDs were converted to official gene symbols according to the Genome Reference Consortium Human Build 38 (GRCh38) assembly. Only genes with transcripts per kilobase of exon model per million mapped reads (TPM) values greater than zero in more than 50% of the samples were included for analysis. The microarray gene expression profiles and clinical information of patients in the GSE21050 dataset (n = 287) were downloaded from the GEO database (https://www.ncbi.nlm.nih.gov/geo/). Patient informed consent exists in these two public datasets, and this study was conducted in accordance with the Helsinki Declaration.

### Discovery and validation of immune subtypes

We applied consensus clustering [55] to identify IMS clusters of patients in the cohort from *TCGA* based on expression of 2006 IRGs (Additional file 4: Table S1). A total of 1914 genes were found in the SARC dataset from *TCGA*. Five hundred bootstraps with 80% item resampling were calculated based on the partition around medoids (PAM) classifier and Euclidean distance. The evaluated K selected for clustering was set as between 2 and 10, and the optimal classification was determined by calculating the consistency matrix and consistency cumulative distribution function. Then, we validated the IMS in the GSE21050 dataset. The in-group proportion (IGP) [56] and *Pearson* correlation among centroids of gene module scores were used to quantitatively measure the consistency and reproducibility of the acquired IMS in the cohorts from *TCGA* and GSE21050. Progression-free survival was calculated from the date of surgery to the date of disease progression (local and/or distal tumor recurrence/metastasis) or to the date of death.

### Evaluation of clinicopathological, molecular, and cellular characteristics associated with IMSs

The PFS period of each sarcoma patient from the two datasets was calculated by using the Kaplan–Meier method with the log-rank test and multivariable Cox regression; samples with survival times less than 30 days were excluded from the analysis. Relationships between the IMS and clinicopathological features, as well as the CINSARC classification, a 67-gene expression signature that stratifies sarcoma prognosis into two subtypes (“low-risk, C1” and “high-risk, C2”) [27], were analyzed by nonparametric (Fisher’s exact) assessments, as appropriate. *TCGA* pancancer study stratifies cancer into six immune subtypes: C1 (wound healing), C2 (IFN-γ dominant), C3 (inflammatory), C4 (lymphocyte depleted), C5 (immunologically quiet), and C6 (TGF-β dominant) [28]. We analyzed relationships between IMSs and *TCGA* classification systems by nonparametric (Fisher’s exact) assessments. To observe the expression profile of chemotherapy-induced immune response-related classic markers [57, 58] and 47 immune checkpoint-related genes from a previous study [59] in the three immune subtypes, we calculated gene expression of the genes in the *TCGA*-SARC and GSE21050 cohorts. To analyze distributions of immune cell components in each IMS, we determined the scores of 28 immune cell types in each patient in the cohorts by analyzing 782 immune cell marker genes [60] using the Single-sample gene set enrichment analysis (ssGSEA) method.

### Defining the immune landscape

Considering the dynamic nature of the immune system, we conducted dimensionality reduction analysis using a graph learning-based method to reveal the intrinsic structure and visualize the distribution of individual patients. Briefly, this analysis projects the high-dimensional gene expression data to a tree structure in a low-dimensional space, where the local geometric information is preserved [61]. This discriminative dimensionality reduction with trees (DDRTree) approach was previously used to model cancer progression and define developmental trajectory using bulk and single-cell gene expression data [62, 63]. Here, we extend the analysis to immune gene expression profiles. This immune landscape reflects the relationship among patients in a nonlinear manifold, which may complement the discrete immune subtypes defined in the linear Euclidean space. The intracluster heterogeneity within each IMS was assessed in terms of gene module expression with ANOVA.

### Analysis of the immune-related gene coexpression module

The WGCNA coexpression algorithm was used to detect coexpressed gene modules in the R package WGCNA [64]. It has been shown that when the logarithm of the connectivity degree node log(k) is negatively related to the logarithm of the probability of emergence of the node log(P(k)) and the correlation coefficient is greater than 0.85, the coexpression network can be a scale-free network [65]. To ensure that the coexpression network was a scale-free network, coexpression modules were screened by setting soft threshold power *β* to 10 (Additional file 2: Fig. S2C). The topology overlap matrix (TOM) was then constructed from the adjacency matrix to avoid influences of noise and spurious associations. Based on TOM, average-linkage hierarchical clustering using the dynamic shear tree method was subsequently conducted to define coexpression modules, and the minimum gene size of each module was set as 60. The feature vector values (eigengenes) of each module were calculated in turn to explore the relationship among modules, and then modules with highly correlating eigengenes were merged into new modules by performing cluster analysis with the following thresholds: height = 0.25, DeepSplit = 4, and minModuleSize = 60.

### Identification of an immune‐related prognostic signature

Univariate Cox regression analysis was performed to determine immune-related gene coexpression modules with prognostic significance. Then, the Cox proportional hazards model, which was suitable for high‐dimensional regression analysis, was used to construct an optimal and prognostic gene set in immune-related modules (package glmnet) [66–68]. Multivariate Cox regression survival analysis was performed to construct the prognostic risk model. The risk score of each patient in the training set was calculated with the linear combination of the gene expression signature weighted by the regression coefficients as follows: Risk score = (exprgene1 × coefficientgene1) + (exprgene2 × coefficientgene2) +$\cdots$+ (exprgenen × coefficientgenen).

### Bioinformatics analysis

The proportions of six tumor-infiltrating immune cell types (B cells, CD4^+^ T cells, CD8^+^ T cells, neutrophils, macrophages, neutrophils and dendritic cells) were estimated using the “Tumor Immune Estimation Resource” (TIMER, https://cistrome.shinyapps.io/timer/) tool [69]. Gene Ontology (GO) enrichment analysis was performed with the R package clusterProfiler. An FDR cutoff value of 0.05 was applied in this test.

### Statistical analysis

All statistical analyses were performed using R 3.6.0 (https://mirrors.tuna.tsinghua.edu.cn/CRAN/) with default software parameters. A P value < 0.05 was considered statistically significant. The biological functions of IRGs in each immune gene coexpression module were annotated in GO using the Tool Database for Annotation, Visualization and Integrated Discovery (DAVID, V6.8). The association between the IMS and all kinds of immune-related molecular and cellular characteristics was assessed using one-way *ANOVA*.

## Supplementary Information

**Additional file1**. **Figure S1**: A-B Cumulative distribution function curve (A) and (B) delta area of immune-related genes in the cohort from *TCGA*.

**Additional file2**. **Figure S2**: A Sample clustering. B Scale-free fit index for various soft-thresholding powers (β). C Mean connectivity for various soft-thresholding powers.

**Additional file3**. **Figure S3**: The estimated proportion of significant immune-related features among IMS subgroups with FDR < 0.05. The top and bottom of the box are the upper quartile (Q3) and the lower quartile (Q1) of the data, respectively. The solid black line in the box represents the median. The whiskers represent the maximum and minimum values of this group of data. The Kruskal-Wallis test was used to assess significant differences. * P < 0.05, ** P < 0.01, *** P < 0.001, and **** P < 0.0001.

**Additional file4**. **Table S1**: List of 2006 immune-related genes.

**Additional file5**. **Table S2**: List of 549 genes with mutation frequency >3 in all subtypes.

**Additional file6**. **Table S3**: List of 86 genes with significantly high mutation frequencies.

**Additional file7**. **Table S4**: The height of each sample in 7 coexpression modules.

**Additional file8**. **Table S5**: List of six prognostic genes in the blue module.

## Data Availability

The datasets generated and analyzed during the current study are available in the *TCGA* repository (https://portal.gdc.cancer.gov/) and the GEO repository (GSE21050, https://www.ncbi.nlm.nih.gov/geo/query/acc.cgi?acc=GSE21050).

## Abbreviations

APCs: Antigen presenting cells
CINSARC: Complexity index in sarcomas
DAVID: Database for annotation, visualization and integrated discovery
DDLPS: Dedifferentiated liposarcoma
DDRTree: Discriminative dimensionality reduction with trees
GEO: Gene Expression Omnibus
GRCh38: Genome Reference Consortium Human Build 38
ICD: Immunogenic cell death
IGP: In-group proportion
IMSs: Immune subtypes
IRGs: Immune-related genes
LMS: Leiomyosarcoma
MLPS: Myxoid liposarcoma
mRNA: Messenger RNA
PC: Principal component
PFS: Progression-free survival
SARC: Sarcoma
SS: Synovial sarcoma
*TCGA*: The Cancer Genome Atlas
TMB: Tumor mutation burden
TME: Tumor microenvironment
TOM: Topology overlap matrix
TPM: Transcripts per kilobase of exon model per million mapped reads
UPS: Undifferentiated pleomorphic sarcoma
WGCNA: Weighted gene correlation network analysis
