# Supplementary figures and images for: The immune subtypes and landscape of sarcomas
Source: BMC Immunol. 2022 Sep 24;23:46. doi: 10.1186/s12865-022-00522-3 (PMC9508767; doi:10.1186/s12865-022-00522-3)

A

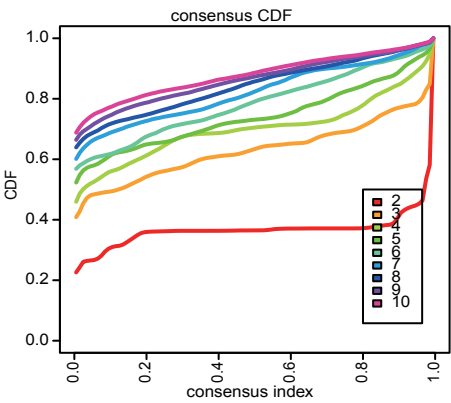

B

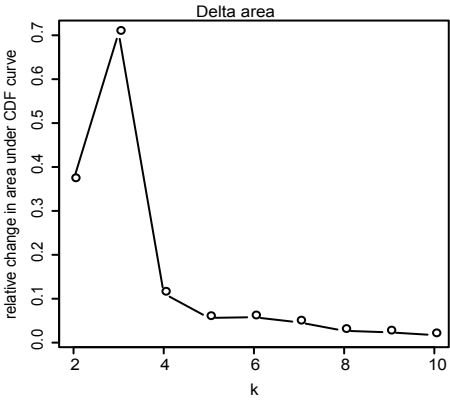

Supplement: Supplementary file 1 — Additional file1. Figure S1: A-B Cumulative distribution function curve (A) and (B) delta area of immune-related genes in the cohort from TCGA. [file 12865_2022_522_MOESM1_ESM.pdf]

Supplementary Fig. 2

A

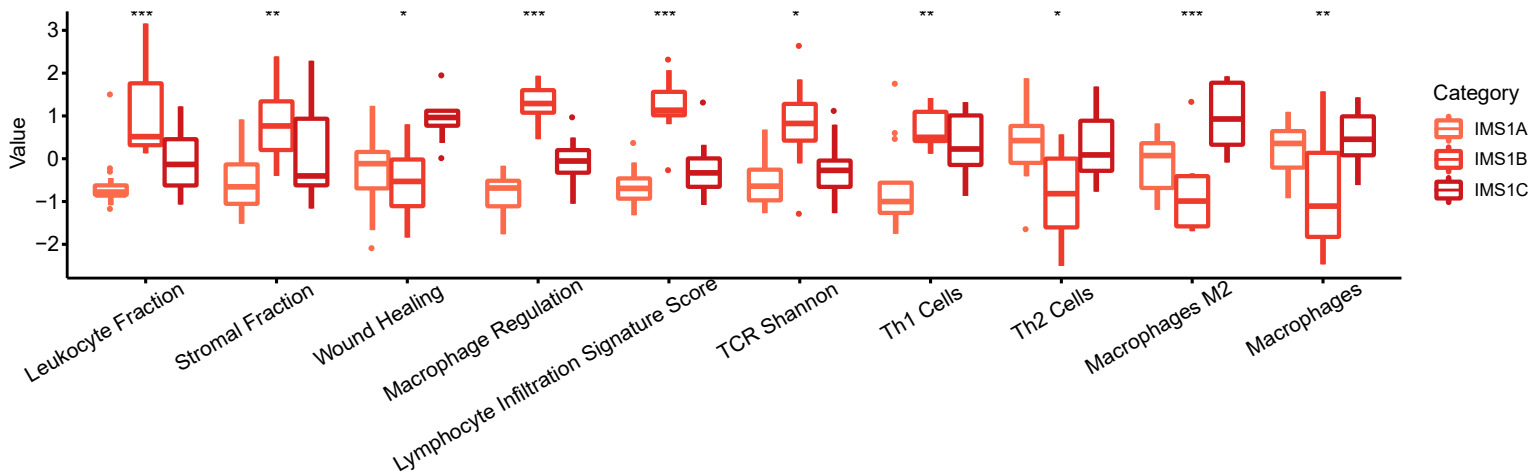

B

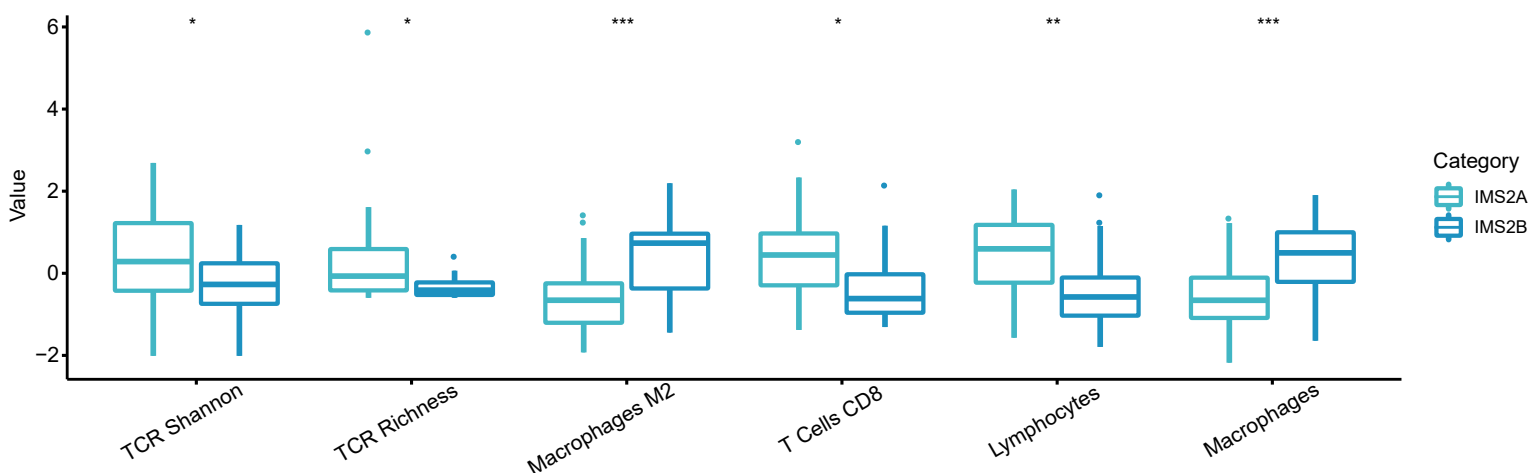

C

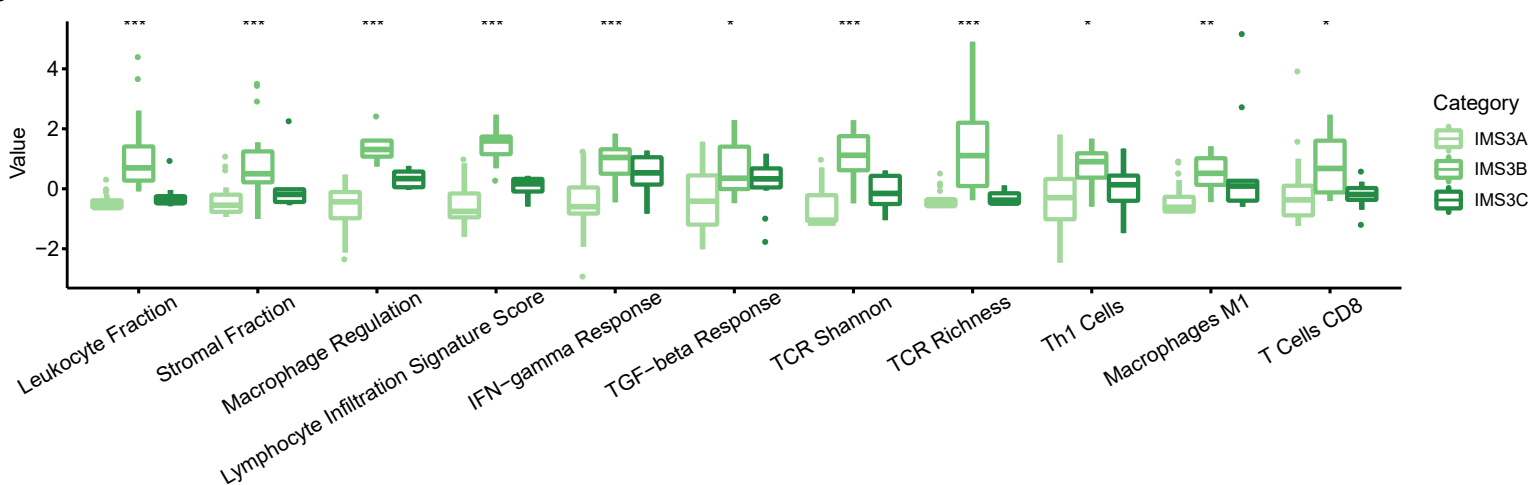

Supplement: Supplementary file 2 — Additional file2. Figure S2: A Sample clustering. B Scale-free fit index for various soft-thresholding powers (β). C Mean connectivity for various soft-thresholding powers. [file 12865_2022_522_MOESM2_ESM.pdf]

A

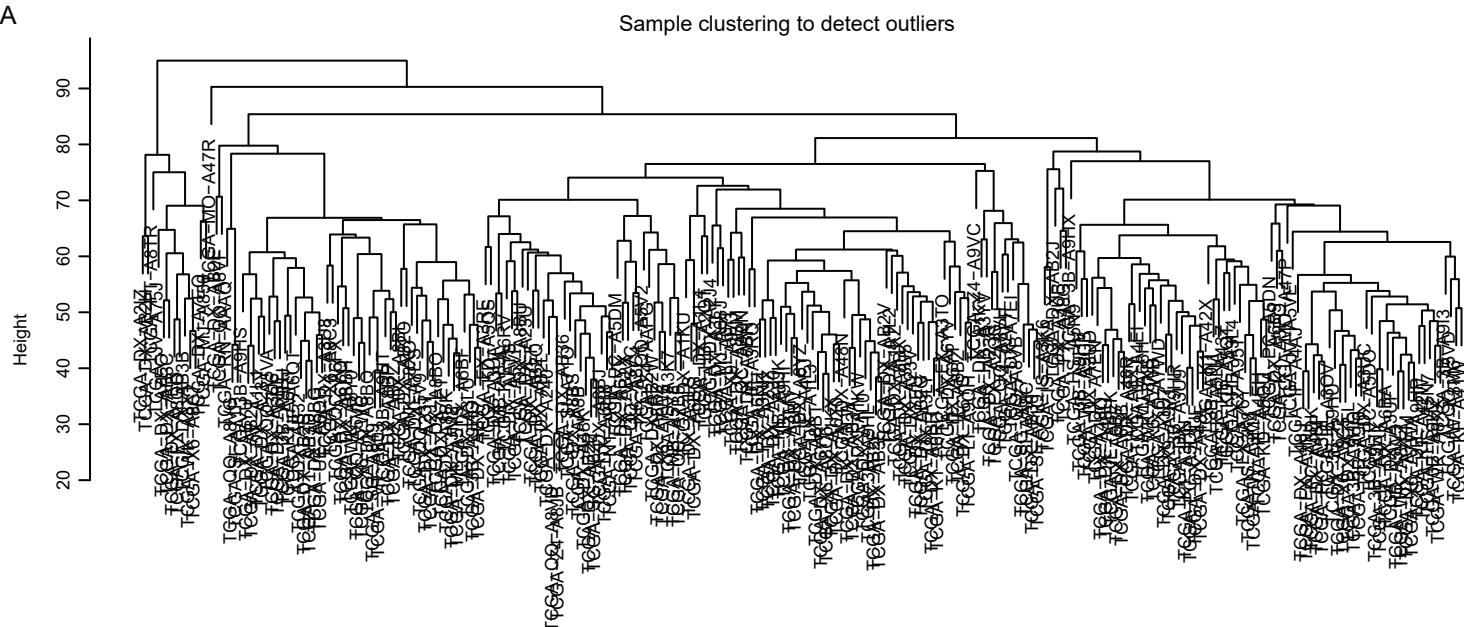

B

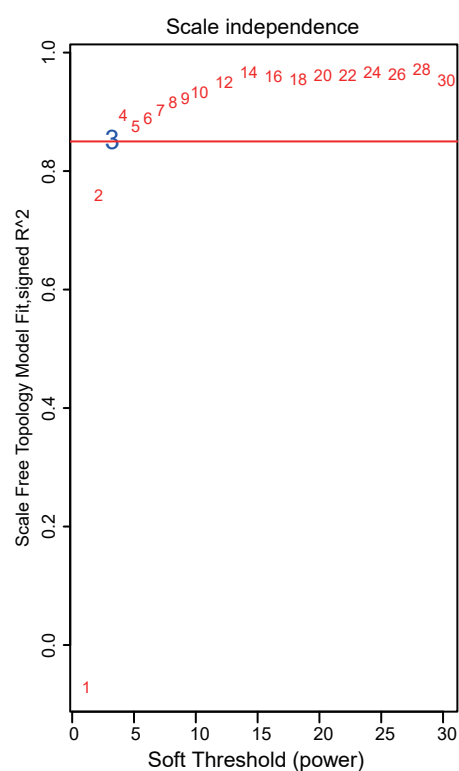

C

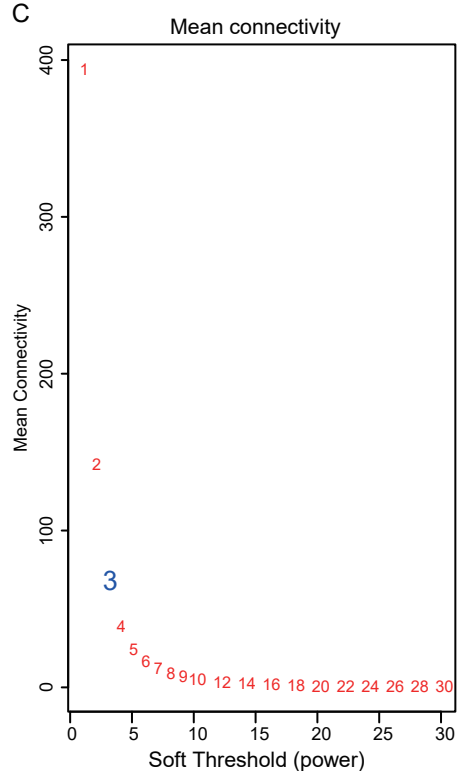

Supplement: Supplementary file 3 — Additional file3. Figure S3: The estimated proportion of significant immune-related features among IMS subgroups with FDR < 0.05. The top and bottom of the box are the upper quartile (Q3) and the lower quartile (Q1) of the data, respectively. The solid black line in the box represents the median. The whiskers represent the maximum and minimum values of this group of data. The Kruskal-Wallis test was used to assess significant differences. * P < 0.05, ** P < 0.01, *** P < 0.001, and **** P < 0.0001. [file 12865_2022_522_MOESM3_ESM.pdf]
